# Epidemiology, real-world treatment and mortality of patients with status epilepticus in Germany: insights from a large healthcare database

**DOI:** 10.1093/braincomms/fcad145

**Published:** 2023-04-30

**Authors:** Antje Mevius, Lars Joeres, Patrick Gille, Manuela Molzan, Nadia Foskett, Thomas Wilke, Ulf Maywald, Felix Rosenow, Adam Strzelczyk

**Affiliations:** Ingress-health HWM GmbH, Real World Evidence department, 23966 Wismar, Germany; UCB Pharma, Neurology, 40789 Monheim, Germany; UCB Pharma, Neurology, 40789 Monheim, Germany; UCB Pharma, Neurology, 40789 Monheim, Germany; UCB Celltech, Real World Evidence global, Slough SL1 3WE, UK; Institut für Pharmakoökonomie und Arzneimittellogistik e.V., University of Wismar, 23966 Wismar, Germany; AOK PLUS, Pharmaceuticals department, 01067 Dresden, Germany; Epilepsy Center Frankfurt Rhine-Main, Goethe-University, 60590 Frankfurt, Germany; Epilepsy Center Frankfurt Rhine-Main, Goethe-University, 60590 Frankfurt, Germany

**Keywords:** status epilepticus, mortality, incidence, burden of disease

## Abstract

Status epilepticus is a life-threatening emergency, and to date, few studies have reported on its long-term treatment and outcomes. This study aimed to estimate the incidence, the treatment and outcomes, the healthcare resource utilization and the costs of status epilepticus in Germany. Data from 2015 to 2019 were obtained from German claims (AOK PLUS). Patients with ≥1 status epilepticus event and no event in the preceding 12 months (baseline) were included. A subgroup of patients with an epilepsy diagnosis during baseline was also analysed. Of the 2782 status epilepticus patients (mean age = 64.3 years; 52.3% female), 1585 (57.0%) were previously diagnosed with epilepsy. The age- and sex-standardized incidence was 25.5 cases/100 000 persons in 2019. The mortality rate after 12 months was 39.8% overall (19.4% and 28.2% after 30 and 90 days, respectively) and 30.4% in the epilepsy patient subgroup. Factors associated with higher mortality were age, comorbidity status, presence of brain tumours and an acute stroke. An epilepsy-related hospitalization at onset of or 7 days prior to the status epilepticus event as well as prescription of antiseizure medication during baseline was associated with a better survival rate. Overall, 71.6% of patients (85.6% in the epilepsy subgroup) were prescribed with out-patient antiseizure medication and/or rescue medication within 12 months. All patients sustained on average 1.3 status epilepticus–related hospitalizations (20.5% had more than one) during a mean follow-up period of 545.2 days (median 514 days); total direct costs including in-patient and out-patient status epilepticus treatments were 10 826€ and 7701€ per patient-year overall and for the epilepsy patient subgroup, respectively. The majority of status epilepticus patients received an out-patient treatment in line with epilepsy guidelines, and patients previously diagnosed with epilepsy have a higher likelihood to receive it. The mortality in the affected patient population is high; risk factors were older age, higher comorbidity burden, the presence of brain tumours or an acute stroke.

## Teaser

Mevius *et al*. studied 2782 patients with status epilepticus based on German claims data. The incidence was 25.5 cases/100 000 in 2019. The mortality rate after 12 months was 39.8%, and 71.6% of patients were prescribed with out-patient antiseizure/rescue medication in line with epilepsy guidelines. Total direct costs were 10 826€ per patient-year.

## Graphical Abstract

**Graphical Abstract:**
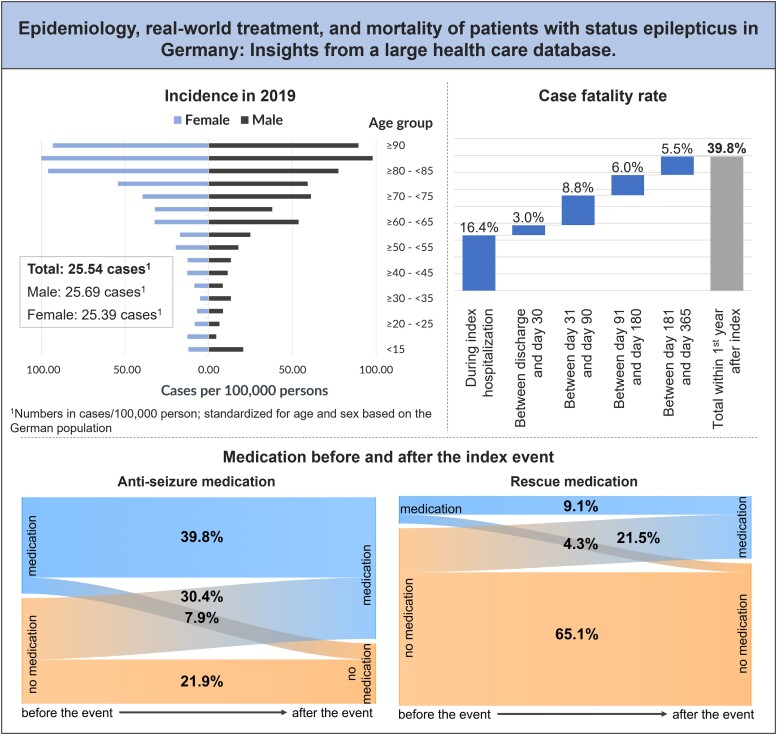

## Introduction

Status epilepticus (SE) is a life-threatening neurological emergency requiring prompt diagnosis and treatment.^1,2^ It describes a prolonged seizure or a series of seizures without regaining consciousness, which may occur in patients with an established diagnosis of epilepsy or in acute or remote disorders of the CNS.^3^ Common causes of SE include ischaemic or haemorrhagic strokes, brain trauma and brain tumours, alcohol abuse, infections and low levels of antiseizure medication (ASM) in patients with epilepsy.^2-7^ The reported incidence of SE in Germany is 15–23/100 000 persons, depending on the underlying populations and methodologies.^7-9^ Long-term consequences can include neuronal injury and alteration of neuronal networks, depending on the type and duration of seizures, with severe associated disabilities.^2,10^ SE is also associated with significant mortality.^2,9^ Reported case fatality rates in population-based studies in Europe varied between 6.6 and 24.4%.^11,12^ According to the guidelines on SE, the treatment of SE includes three steps: (i) benzodiazepines as first-line medication of choice [usually as rescue medication (RM) by laypeople or by paramedics in out-patient setting or a first in-patient treatment]; (ii) ASM if benzodiazepines are not effective or to ensure and maintain long-term therapeutic success (mainly levetiracetam or valproic acid, both are not or only limited approved for SE); and (iii) anaesthetics for treatment of refractory patients (continuous midazolam, propofol or thiopental).^12,13^ The general aims of treating a patient in SE are to achieve rapid control of seizures and avoid life-threatening complications.^12,14^

Representative data addressing long-term mortality, pharmaceutical treatment, explicit treatment with ASM and characteristics of patients during in-patient treatment due to and after SE are missing.^15^ The aim of this study was to estimate the incidence of SE in 2019 in Germany; to describe the characteristics and real-world treatment of patients after discharge from an incident SE hospitalization, their mortality and related risk factors for early death; and to assess the associated healthcare resource use (HCRU) and costs.

## Materials and methods

### Study design

This retrospective cohort study was based on anonymized claims data covering the period from 1 January 2015 to 31 December 2019, provided by the regional German statutory health insurance fund AOK PLUS (the year 2020 was excluded from the observation to avoid potential unknown effects due to COVID-19). This dataset covers ∼3.4 million individuals from the German federal states of Saxony and Thuringia, corresponding to ∼4% of the German population and 4.7% of the German population insured by statutory health insurance. A study site or selection bias does not exist due to the nature of the data. The general study design and a description of the dataset were published previously.^16-19^

German claims data provide information on patient demographics (age, sex and date of death) and detailed reimbursement claims on out-patient care, in-patient care, pharmaceuticals, therapeutic devices, rehabilitation and sick leaves. The out-patient care data comprise information on diagnostic and therapeutic procedures {according to the German Uniform Valuation Scheme [Einheitlicher Bewertungsmaßstab (EBM)]}^20^ and the diagnosis made by an out-patient physician as well as the type of treating physician (identified by the physician code, the ‘Arztgruppenschlüssel’). The in-patient care data cover information on the date of admission and length of stay and diagnostic and therapeutic procedures [according to the operation and procedure coding, the Operationen und Prozedurenschlüssel (OPS)].^21^ In-patient and out-patient diagnoses are coded according to the German Modification of the International Classification of Diseases 10th Revision (ICD-10-GM). Data on out-patient prescriptions of reimbursed drugs include information on the date of prescription, the type of prescribing physician and the pharmaceutical reference number of the prescribed agents. The reference number is linked to information on the Anatomical Therapeutic Chemical classification code (ATC),^22^ the defined daily dose, the packaging size, the strength and the formulation of the drug.^16-19^

### Study population

#### Newly diagnosed SE population

All included patients experienced at least one in-patient hospitalization with a diagnosis of SE (main or secondary; ICD-10 G41.-) in the period 1 January 2016–31 December 2018 (first observed hospitalization = index date), had a 12-month period without any SE-related hospitalization prior to the index date and were continuously insured by the health insurance from 12 months before until 12 months after the index date or death (whatever occurred first). SE is one of the life-threatening conditions, especially for patients with epilepsy; therefore, as a subgroup of all SE patients, those with at least one in-patient or out-patient epilepsy diagnosis (ICD-10 G40.-) in the 12 months before the index date were analysed separately.

#### Population with incident SE in 2019

In addition, for the assessment of the most recently available incidence, patients with an incident SE diagnosis in 2019 were observed separately. The denominator for the incidence calculation was all in the AOK PLUS insured persons in 2019 alive on 1 January 2019, being continuously insured in the year 2018.

### Study outcomes and analyses

#### Incidence in 2019

In this analysis, patients were only included if they experienced at least one hospitalization with a diagnosis of SE (main or secondary; ICD-10 G41.-) in the period 1 January 2019–31 December 2019, with a 12-month period without any SE-related hospitalization prior to the index date and with continuous insurance for 12 months before the index date (subgroup of all above incident SE patients).

The number of identified SE patients in 2019 was divided by all insured patients in that year alive on 1 January 2019, being continuously insured in the year 2018. Age- and sex-specific incidences were reported as identified SE cases in 2019 per 100 000 persons in 5-year-age/sex groups. The assignment to an age group was based on the age of a patient at the index date. The extrapolation of incidence numbers to the total population was based on the statistics for the current population of Germany from the Federal Statistical Office in Germany.^23^

#### Patient characteristics

In all the following analyses, patients were observed starting with their first SE-related hospitalization (index date from 1 January 2016 to 31 December 2018) until 31 December 2019 or end of insurance or death (whatever came first).

Patient characteristics for the overall incident SE population were descriptively analysed based on the respective index date (date of first observed SE hospitalization) or a 12-month pre-index period. In addition to age and sex, the comorbidity status [using the Charlson Comorbidity Index (CCI)]^24^ was observed. The type of the incident SE event was analysed based on the fourth digit of the ICD-10 code: G41.0 grand mal, G41.1 petit mal, G41.2 complex partial and G41.8/G41.9 other or unspecified SE. SE aetiology was reported, in line with Knake *et al*.,^7^ as the occurrence of at least one hospitalization with one of the following main diagnoses in 7 days preceding the index date (acute symptomatic): stroke (ICD-10 I60-I64), progressive vascular leucoencephalopathy (ICD-10 I67.3), brain tumour (ICD-10 C71), any metabolic disorder (ICD-10 E70-E90), mental or behavioural disorders due to use of alcohol (ICD-10 F10) or injuries to the head (ICD-10 S00-S09). Factors were classified as remote symptomatic if respective hospitalizations occurred within the 12-month baseline period but >7 days before the index date.^7^ The occurrence of an epilepsy-related hospitalization in the 12-month baseline period was classified as potentially causing the SE event—a low drug level according to Knake *et al*.^7^ could not be captured by the data. A patient could have been diagnosed with more than one of the SE-causing conditions.

#### Case fatality

Case fatality after the index event was assessed by means of Kaplan–Meier analysis, censoring for end of insurance or end of data availability (31 December 2019), whatever came first. Median survival time and the proportion of patients who died after 1, 3, 6, 12 and 24 months were reported. A multivariable Cox regression model assessed risk factors for early death, taking into account available patient characteristics such as age, sex and CCI as well as aetiology of SE and use of ASM in the baseline period.

#### SE-related pharmacological treatment

The number of patients who received at least one prescription of ASM and/or RM (identified via ATC; Supplementary Table 1) in the 12 months before and after the index date was reported. Additionally, we calculated the number of patients who did not receive any ASM and/or RM during the baseline and entire follow-up periods; in a sensitivity analysis, this included only those patients who survived at least the first 30 days after the index SE event.

#### HCRU and costs

HCRU was assessed in terms of the number of patients with at least one SE- or epilepsy-related out-patient visit [specialists and general practitioners (GP), approximated by counted dates of invoiced codes according to the uniform valuation scheme (EBM)] and the related number of visits per observed patient/patient-year, as well as the number of SE-related hospitalizations per patient/patient-year, the number of days in the hospital per patient-year and the number of out-patient prescriptions (identified via ATC). Additionally, SE-related direct costs for out-patient visits [ICD-10 G40/G41; calculated as the number of visits with points per visit, which express the complexity of a visit according to a German point value system (EBM)], hospitalizations (ICD-10 G41; costs paid by the health insurance as documented in the database) and prescribed ASM and RM (calculated with pharmacy sales prices of each medication) following the index event were reported per observed patient-year. Additionally, hospitalization costs were reported per observed in-patient hospitalization.

#### Statistical analysis

For all categorical variables, the number and percentage of patients in each category were reported. Summary statistics, including mean and standard deviation (SD), were applied for all continuous variables. The Cox regression model was conducted as backward elimination regression, fitting the model by deleting stepwise insignificant variables. Hazard ratios (HR) with 95% confidence intervals (CI) and *P*-values were reported for all significant variables in the model (*P* ≤ 0.05). All statistical analyses were performed using Microsoft SQL Server 2014, STATA/MP 14 and Microsoft Excel (MS 365).

#### Regulatory aspects and general considerations

The analysis was approved by the ethics committee of the University of Frankfurt, and informed consent was waived due to the use of an anonymized dataset. The study protocol was reviewed by a scientific steering committee and the data owner. The study was conducted and reported in accordance with guidelines for secondary data studies.^25-27^ The work on the dataset conformed to all social security data protection requirements.

## Results

### Patient selection

In total, 2782 patients with at least one SE-related hospitalization following a 12-month SE-free period could be identified for the period 2015–18. The mean observational time was 545.2 days for all patients. Among these SE patients, 1585 (57.0%) were coded with at least one in-patient or out-patient epilepsy diagnosis in the 12-month period prior to the index event. The mean observational time was 635.8 days in this subgroup. Reported types (according to the ICD-10 definition; more than one diagnosis per hospitalization were possible) of the incident SE were complex partial SE (ICD-10 G41.2; 34.2%), grand mal SE (ICD-10 G41.0; 22.2%), petit mal SE (ICD-10 G41.1; 3.7%) and other/unspecified SE (ICD-10 G41.8/G41.9; 48.5%).

### Incidence in 2019

**Figure 1:**
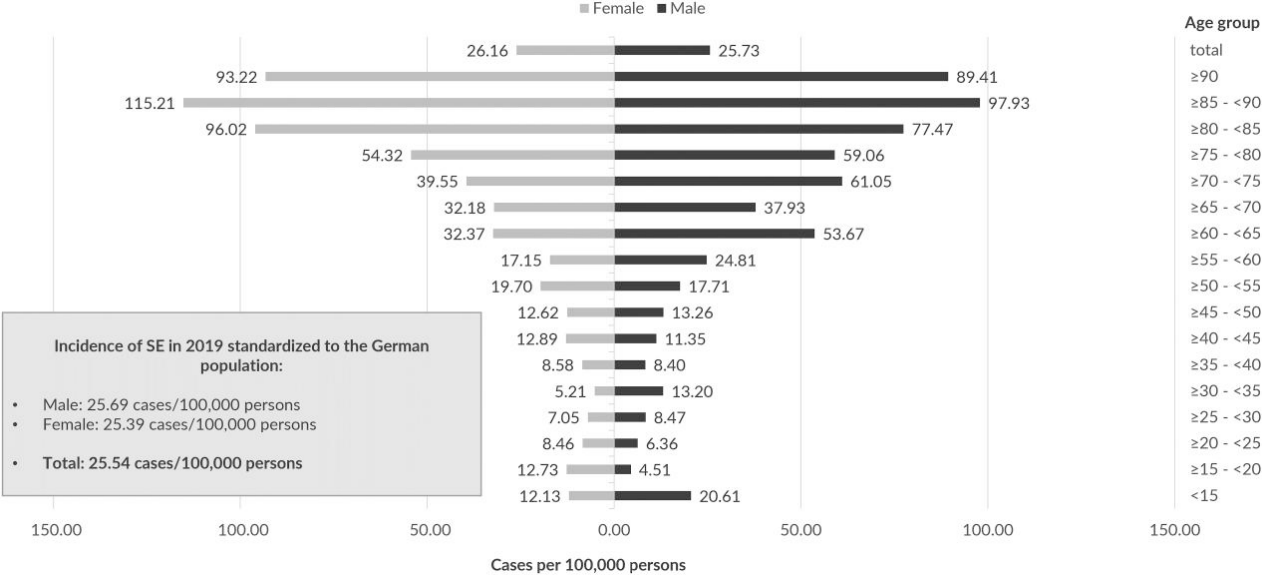
**Incidence of SE cases in 2019** per 100 000 persons in 5-year-age/sex groups; extrapolated by age and sex standardization to the total population of Germany.

For the year 2019, 930 patients of 3.2 Mio insured were identified with at least one incident SE-related hospitalization after a 12-month period with continuous insurance and without any previous SE-related hospitalizations in a 12-month baseline period. The incidence of SE (in cases/100 000 persons) was 29.1 cases in the AOK PLUS population and 25.5 standardized to the German population (Fig. 1). The overall incidence rate was nearly identical for the male and female population, but in the older age groups, the incidence in female persons was higher (≥80−<85 years: 96.0 versus 77.5), whereas in the younger population, the SE incidence in male persons was higher (<15 years: 20.6 versus 12.1).

### Patient characteristics

Based on the sample included on 1 January 2016–31 December 2018, SE patients had a mean age of 64.3 years (SD 23.4), and the proportion of female patients was 52.3%; patients with epilepsy at baseline were slightly younger with a mean age of 62.3 years and 49.9% being female. The mean CCI in the baseline period was nearly identical in the overall SE population and the subgroup with a previous epilepsy diagnosis (4.42 and 4.43) (Table 1).

**Table 1:** Baseline characteristics of identified SE patients

	All SE patients	SE patients with an epilepsy diagnosis at baseline
Number of patients	2782	1585
Mean (SD│median) observation time in days	545.2 (456.1│514)	635.8 (442.6│631)
Mean (SD│median) age in years at the index date	64.3 (23.4│70.5)	62.3 (22.6│66)
Male/female gender	47.7%/52.3%	50.1%/49.9%
Mean (SD│median) CCI for the 12-month pre-index period	4.4 (3.5│4)	4.4 (3.6│4)
*Etiologic factors*		
Stroke		
*n* (%) acute^a^	212 (7.6%)	57 (3.6%)
*n* (%) remote^b^	247 (8.9%)	156 (9.8%)
Progressive vascular leucoencephalopathy		
*n* (%) acute^a^	0	0
*n* (%) remote^b^	1 (0.1%)	0
Brain tumour		
*n* (%) acute^a^	15 (0.5%)	6 (0.4%)
*n* (%) remote^b^	17 (0.6%)	10 (0.6%)
Metabolic disorder		
*n* (%) acute^a^	21 (0.8%)	10 (0.6%)
*n* (%) remote^b^	103 (3.7%)	65 (4.1%)
Mental or behavioural disorders due to use of alcohol		
*n* (%) acute^a^	18 (0.7%)	9 (0.6%)
*n* (%) remote^b^	75 (2.7%)	52 (3.3%)
Injuries to the head		
*n* (%) acute^a^	59 (2.1%)	18 (1.1%)
*n* (%) remote^b^	113 (4.1%)	77 (4.9%)
Epilepsy-related hospitalization		
*n* (%) in the 12-month baseline period	472 (17%)	472 (29.8%)

a Acute: at index hospitalization or respective in-patient main diagnosis within 7 days before the index date. ^b^Remote: respective in-patient main diagnosis within the 12-month baseline period but >7 days before index hospitalization.

In all SE patients, the most observed underlying aetiologic factors for the occurrence of SE were hospitalization with epilepsy (17.0%), stroke (remote 8.9% and acute 7.6%) and head injuries (remote 4.1% and acute 2.1%) (Table 1). For 51.6% of all SE patients, aetiology was unknown or different from the defined potential factors.

### Case fatality

In total, 1378 (49.5%) of all SE patients died during the whole follow-up period and 666 (42.0%) of patients with epilepsy that was diagnosed before the incident SE episode. Median survival was 929 days for all SE patients (61 days survival time for 25%), and >50% of those with a previous epilepsy diagnosis survived till end of the observation (median not reached; 207 days for 25%).

**Figure 2:**
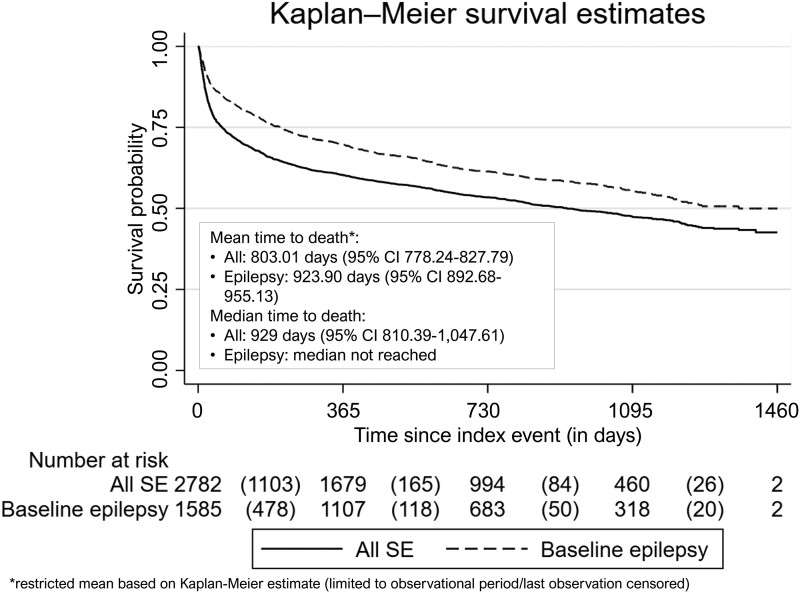
**Overall survival for all SE patients and for patients with epilepsy at baseline**; Kaplan–Meier curve, restricted mean and median time to death based on Kaplan–Meier estimate (95% CI).

The number of dead patients/case fatality rate after 1 month was 540 patients/19.4%, after 3 months 785/28.2%, after 6 months 953/34.3% and after 12 months 1106/39.8%. After 24 months, the Kaplan–Meier-based mortality rate taking into account censoring as described above was 46.6% (Fig. 2). The patients who died during follow-up were on average 75.2 years, and the proportion of female patients was 57.4%. The overall 1-year mortality rate after the index date per age group increased from 6.5% in patients aged 30−<40 years by 10–15 percentage points per life decade up to 72.1% in the highest age group ≥90 years.

**Figure 3:**
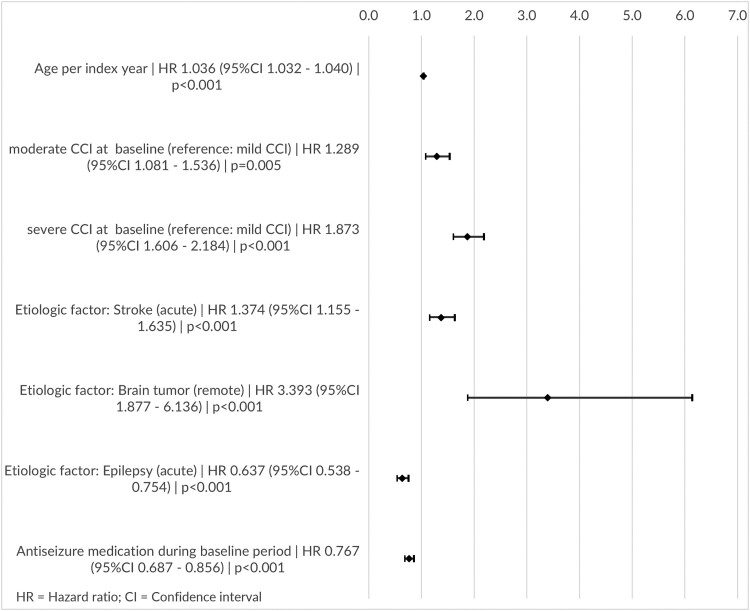
**Estimated death risks of impact factors**; results from the multivariable Cox regression model for time to death: HR (95% CI) and *P*-values.

The multivariable Cox regression model revealed two variables associated with a lower probability of early death: an epilepsy-related hospitalization within 7 days before the SE event or at the date of the SE event (HR 0.637; 95% CI: 0.538–0.754) and the prescription of ASM during the baseline period (HR 0.767; 95% CI: 0.687–0.856). Factors associated with a higher risk of early death were higher age (HR 1.036 per year; 95% CI: 1.032–1.040), moderate CCI (index 3–4; HR 1.289; 95% CI: 1.081–1.536) or severe CCI (index >4; HR 1.873; 95% CI: 1.606–2.184), an acute stroke diagnosis within 7 days before the SE event or at the date of the SE event (HR 1.374; 95% CI: 1.155–1.635) and the presence of a brain tumour diagnosis during the baseline period (HR 3.393; 95% CI: 1.877–6.136) (Fig. 3). Variables excluded from the model because of insignificance (*P* > 0.05) were sex, remote stroke, acute or remote metabolic disorder, remote or acute alcohol-related disorder, acute or remote brain injury and epilepsy-related hospitalizations in the 12-month baseline period.

### SE-related pharmacological treatment

Among all SE patients, 1326 (47.7%) received an ASM and 373 (13.4%) had a RM prescription during the baseline period before the incident SE episode, with levetiracetam (25.3%), valproic acid (13.1%) and lamotrigine (8.1%) as the most frequently prescribed ASM agents and with lorazepam (5.6%), diazepam (5.2%) and midazolam (1.8%) as the most frequently prescribed RM agents. Among the patients with an epilepsy diagnosis at baseline, 1176 (74.2%) received an ASM and 302 (19.1%) were prescribed a RM before the index hospitalization.

In the first 12 months of the follow-up period, 1920 (69.0%) of all SE patients and 1332 (84.0%) of the patients with epilepsy at baseline were prescribed an ASM. The most frequently prescribed medications were levetiracetam (47.4%/56.5%), valproic acid (23.2%/30.7%) and lacosamide (13.1%/18.0%). If only patients who survived the first 30 days after the index SE event were observed, 83.6% of all observed SE patients and 92.9% of the patients previously diagnosed with epilepsy received an ASM.

Following the SE episode, 666 (23.9%) were prescribed a RM; this number was higher with 483 (30.5%) among those with an epilepsy diagnosis prior to SE. Most frequently prescribed medications were diazepam (10.2%/12.9%), lorazepam (10.0%/12.2%) and midazolam (4.5%/5.2%) (Table 2). If only patients who survived the first 30 days after the index SE event were observed, 28.5% of all observed SE patients and 33.2% of the patients previously diagnosed with epilepsy received a RM. Among all observed SE patients, 1014 (36.5%) received more than one ASM during the first 12 months after the index date, and 551 patients (19.8%) did not receive any ASM or RM neither in the baseline nor in the first 12 months of follow-up. In the subgroup of patients with epilepsy at baseline, 817 patients (51.6%) were prescribed more than one ASM; the number of patients without any ASM or RM in this group was 74 (4.7%).

**Table 2:** Most frequently prescribed ASM/RM during the 12-month baseline period and the first 12 months of the follow-up period

Observed agents	All SE patients (2782 patients)		SE patients with an epilepsy diagnosis at baseline (1585 patients)	
	Baseline period	12 months of follow-up	Baseline period	12 months of follow-up
ASM				
Levetiracetam	704 (25.31%)	1318 (47.38%)	689 (43.47%)	896 (56.53%)
Valproic acid	363 (13.05%)	645 (23.18%)	353 (22.27%)	486 (30.66%)
Lamotrigine	224 (8.05%)	311 (11.18%)	219 (13.82%)	271 (17.10%)
Pregabalin	136 (4.89%)	93 (3.34%)	71 (4.48%)	51 (3.22%)
Carbamazepine	129 (4.64%)	99 (3.56%)	124 (7.82%)	87 (5.49%)
Gabapentin	115 (4.13%)	72 (2.59%)	66 (4.16%)	49 (3.09%)
Lacosamide	112 (4.03%)	363 (13.05%)	112 (7.07%)	286 (18.04%)
Oxcarbazepine	74 (2.66%)	94 (3.38%)	72 (4.54%)	79 (4.98%)
Zonisamide	54 (1.94%)	107 (3.85%)	54 (3.41%)	88 (5.55%)
Topiramate	50 (1.80%)	69 (2.48%)	48 (3.03%)	57 (3.60%)
Primidon	39 (1.40%)	31 (1.11%)	37 (2.33%)	29 (1.83%)
Other ASM^a^ (<1%)	128 (4.60%)	235 (8.45%)	125 (7.89%)	194 (12.24%)
No ASM	1456 (52.34%)	862 (30.98%)	409 (25.80%)	253 (15.96%)
RM				
Lorazepam	156 (5.61%)	278 (9.99%)	109 (6.88%)	194 (12.24%)
Diazepam	145 (5.21%)	283 (10.17%)	128 (8.08%)	204 (12.87%)
Midazolam	51 (1.83%)	124 (4.46%)	48 (3.03%)	83 (5.24%)
Clobazam	41 (1.47%)	94 (3.38%)	41 (2.59%)	85 (5.36%)
Clonazepam	40 (1.44%)	49 (1.76%)	36 (2.27%)	40 (2.52%)
Other RM^b^ (<1%)	10 (0.36%)	7 (0.25%)	6 (0.38%)	5 (0.32%)
No RM	2409 (86.59%)	2116 (76.06%)	1286 (80.95%)	1102 (69.53%)

a Other ASM with <1% in at least one of the reported periods: eslicarbazepine, phenytoin, perampanel, brivaracetam, phenobarbital, sultiame, ethosuximide, vigabatrin, stiripentol, rufinamide, acetazolamide and potassium bromide. ^b^Other RM with <1% in at least one of the reported periods: alprazolam and chloral hydrate.

### HCRU and costs

Based on all observed SE patients, 55.6% had at least one epilepsy-related out-patient GP visit in the follow-up period, 37.4% visited a neurologist and 31.7% visited another specialist associated with an epilepsy diagnosis. Including the index hospitalization, every patient experienced on average 1.3 SE-related hospitalizations (SD 0.9; 0.9 per patient-year) with a mean length of stay of 13.7 days (SD 17.5; 12.2 days per patient-year) in the whole follow-up period. In the subgroup of SE patients with an epilepsy diagnosis at baseline, this was 1.4 hospitalizations (SD 1.0; 0.8 per patient-year) with a mean length of 10.9 days (SD 14.7; 8.8 per patient-year) (Table 3).

**Table 3:** HCRU and costs during the follow-up period

	All SE patients		SE patients with an epilepsy diagnosis at baseline	
	HCRU	Mean costs per patient-year	HCRU	Mean costs per patient-year
Number of patients/patient-years (py)	2782/4155.2		1585/2761.0	
Epilepsy-related out-patient visits				
Number (%) of patients with GP visits	1547 (55.6%)	373€	1152 (72.7%)	450€
Mean number of GP visits per py	2.5		3.0	
Number (%) of patients with neurologist visits	1041 (37.4%)		809 (51.0%)	
Mean number of neurologist visits per py	1.5		1.9	
Number (%) of patients with visits to other specialists	881 (31.7%)		684 (43.2%)	
Mean number of visits to other specialists per py	1.2		1.5	
SE-related hospitalizations				
Mean number of hospitalizations per py (per patient)	0.9 (1.3)	9288€ (10 467€ per hospitalization)	0.8 (1.4)	5806€ (7208€ per hospitalization)
Mean number of in-patient days per py	12.2		8.8	
Mean (median) length of stay in days	13.7 (8)		10.9 (6)	
Number (%) of patients with more than one hospitalization	569 (20.5%)		377 (23.8%	
Out-patient SE-related medication				
Number (%) of patients with ASM prescriptions	1952 (70.2%)	1133.23€	1347 (85.0%)	1404€
Mean number of ASM prescriptions per py	8.6		10.2	
Number (%) of patients with RM prescriptions	830 (29.8%)	33.02€	620 (39.1%)	41€
Mean number of RM prescriptions per py	0.9		1.1	
Total costs	10 826€		7701€	

Mean direct SE-related costs (including index hospitalization) per observed patient-year were 10 826€ for all patients, with 85.8% of costs resulting from hospitalizations. Mean direct SE-related costs in the subgroup of SE patients pre-diagnosed with epilepsy were 7701€ per patient-year (75.4% due to hospitalizations) (Table 3).

## Discussion

Based on a large German claims dataset, this study evaluated the characteristics, real-world treatment and resource utilization of patients with an incident SE-related hospitalization. The main strength of this study is the size and representativeness of the database, which is unaffected by any site or patient selection bias, which typically limits the generalizability of registries or prospective observational studies. In addition, mortality information is 100% complete in German claims data, which is another advantage in comparison with other databases that often lack this information.

A cohort of 2782 SE patients and a subgroup of 1585 patients with an epilepsy diagnosis in the 12-month period before the index event were identified. The age- and sex-standardized overall incidence of 25.5 cases/100 000 persons is comparable with the results of previously published population-based retrospective studies.^9,28-30^ Epilepsy and stroke in the baseline period were the most common aetiologic factors causing SE in our study. In previous publications, higher rates of remote conditions were reported,^3,7^ which can be mainly explained by our limited baseline period of 12 months and a very restrictive definition of the presence of an aetiologic factor in the present study (a hospitalization with the respective main diagnosis at the date of the SE event and/or in the baseline period).

Our reported case fatality rate at 30 days of 19.4% is comparably high but expected as we obviously observed a comparatively old patient population that suffers from underlying conditions with CNS damage. The risk factors, identified in the multivariable Cox regression model, support this explanation: older age, a previous stroke, a brain tumour and a high comorbidity status are associated with a higher mortality risk. Other studies reported in-hospital/30-day case fatality rates between 10 and 25%, depending on the clinical definition of observed events and the population.^9,29-33^ However, observed patients were typically younger and had fewer comorbidities than in our study.

Most patients received levetiracetam and valproic acid as an out-patient antiseizure treatment following the SE-related hospitalization to prevent subsequent seizures, which is in line with the recommendations from epilepsy and SE guidelines^12,13,34^ and was shown by cohort-based studies from Germany.^35,36^ A substantial part of the 791 (28.4%) SE patients without any ASM or RM during the first 12 months after the index date died within the first 30 days of observation (480 patients). There is a high probability that an ASM treatment of these patients was clinically not feasible, i.e. after acute stroke. Excluding these patients, a proportion of 11.2% of all SE patients remained untreated with ASM. A more in-depth analysis of these patients is recommended, as a missing treatment is not in line with guideline recommendations.^12,34^ In addition, only a quarter of all observed SE patients (23.9%) received at least one prescription of an out-patient RM after index hospitalization, which is comparable with findings from a German multicentre cohort study by Kadel *et al*.^37^

A comparison of our reported cost numbers with previous studies is challenging as most studies published costs of SE hospitalizations only. One of the strengths of our study is that we report the overall direct healthcare costs per observed patient-year (10 826€), which is based on our view the most objective way to report SE costs considering the out-patient treatment and out-patient prescriptions SE patients receive and taking into account that censoring due to early death of some SE patients is frequent.

Nevertheless, hospitalization costs account for the majority of our costs, and we also report mean direct SE-related costs of 10 467€ per hospitalization, which is in line with previous studies. Two German studies reported mean costs of 11 563€ and 14 946€ for a comparable population,^9,36^ while mean admission costs of 15 880€ were higher for a paediatric population.^8^ Our hospitalization costs for patients with an epilepsy diagnosis at baseline were substantially lower (7208€ per hospitalization). This indicates that the causing factors influence the severity of the SE, the length of stay and therefore the costs.^38^

We acknowledge some limitations of our analysis. First, our data did not provide any information about clinical details, such as severity, clinical classification, duration of the SE episode, refractory status or cause of death. However, ICD-10 coding was used in previous publications to identify epilepsy and SE admissions in Germany and other countries.^8,9,29^ Second, due to the German reimbursement system, only very limited information about medication during hospitalizations is available, especially the administration of emergency medication as in-patient treatment could not be ascertained. Third, we used the AOK PLUS dataset, which contains 3.4 million patients representing 4.7% of the German statutory health insurance population and covering two large regions of Germany (Saxony and Thuringia). Therefore, a regional bias of the study cannot be ruled out completely. Nevertheless, since treatment patterns and reimbursement rules for statutory health insurance are comparable across Germany, the representativeness of the dataset concerning the diagnosis and treatments is likely.

## Conclusion

The incidence of SE observed was 25.5/100 000. Our study indicates that the real-world treatment of patients with an SE event is generally in line with guideline recommendations. The most frequently observed antiseizure treatments were levetiracetam and valproic acid. Nevertheless, we observed a minor proportion of untreated patients. The overall mortality in SE patients is very high with 39.8% in the first year after the SE event; >50% of fatalities occurred within the first 3 months after the incident SE. Our study identified mean SE-related costs of 10 826€ per patient-year—mainly caused by hospitalization costs.

## Supplementary Material

fcad145_Supplementary_Data

## Data Availability

Data from non-interventional studies are outside of UCB’s data sharing policy and are unavailable for sharing.

## Abbreviations

ASM =: antiseizure medication
ATC =: Anatomical Therapeutic Chemical classification code
CCI =: Charlson Comorbidity Index
CI =: confidence interval
EBM =: Einheitlicher Bewertungsmaßstab
GP =: general practitioner
HCRU =: healthcare resource use
HR =: hazard ratio
ICD-10-GM =: German Modification of the International Classification of Diseases 10th Revision
OPS =: Operationen und Prozedurenschlüssel
RM =: rescue medication
SD =: standard deviation
SE =: status epilepticus
