# Supplementary material for: Epidemiology, real-world treatment and mortality of patients with status epilepticus in Germany: insights from a large healthcare database
Source: Brain Commun. 2023 Apr 30;5(3):fcad145. doi: 10.1093/braincomms/fcad145 (PMC10174205; doi:10.1093/braincomms/fcad145)
Supplement: fcad145_Supplementary_Data [file fcad145_supplementary_data.docx]

Supplemental Table 1: Antiseizure medication (ASM) and rescue medication (RM) available in Germany

| **Active substance** | **Classification** | **Identification (ATC code)** |
| --- | --- | --- |
| Acetazolamide | ASM | S01EC01 |
| Brivaracetam | ASM | N03AX23 |
| Carbamazepine | ASM | N03AF01 |
| Eslicarbazepine | ASM | N03AF04 |
| Ethosuximide | ASM | N03AD01 |
| Felbamate | ASM | N03AX10 |
| Fosphenytoin | ASM | N03AB05 |
| Gabapentin | ASM | N03AX12 |
| Lacosamide | ASM | N03AX18 |
| Lamotrigine | ASM | N03AX09 |
| Levetiracetam | ASM | N03AX14 |
| Oxcarbazepine | ASM | N03AF02 |
| Perampanel | ASM | N03AX22 |
| Phenobarbital | ASM | N03AA02 |
| Phenytoin | ASM | N03AB02 |
| Potassium bromide | ASM | N03AX31 |
| Pregabalin | ASM | N03AX16 |
| Primidon | ASM | N03AA03 |
| Retigabine | ASM | N03AX21 |
| Rufinamide | ASM | N03AF03 |
| Stiripentol | ASM | N03AX17 |
| Sultiame | ASM | N03AX03 |
| Tiagabine | ASM | N03AG06 |
| Topiramate | ASM | N03AX11 |
| Valproic acid | ASM | N03AG01 |
| Vigabatrin | ASM | N03AG04 |
| Zonisamide | ASM | N03AX15 |
| Alprazolam | RM | N05BA12 |
| Chloral hydrate | RM | N05CC01 |
| Clobazam | RM | N05BA09 |
| Clonazepam | RM | N03AE01 |
| Diazepam | RM | N05BA01 |
| Lorazepam | RM | N05BA06 |
| Midazolam | RM | N03AE03 / N05CD08 |
